# Basic Periodontal Treatment Improves Periodontal Inflamed Surface Area Without Adverse Events in Patients Receiving Direct Oral Anticoagulants: A Retrospective Clinical Study with an Animal Model

**DOI:** 10.3390/biomedicines14091953

**Published:** 2026-08-30

**Authors:** Ryousuke Fujimori, Yuri Taniguchi, Kazuhisa Ouhara, Shunsuke Miyauchi, Shinji Matsuda, Naoya Kuwahara, Shoya Ueda, Yitong Hou, Masaru Shimizu, Misako Tari, Shoko Kono, Tomoyuki Iwata, Tomoaki Shintani, Mikihito Kajiya, Mutsumi Miyauchi, Yukiko Nakano, Noriyoshi Mizuno

**Affiliations:** 1 Department of Periodontal Medicine, Graduate School of Biomedical and Health Sciences, Hiroshima University, 1-2-3 Kasumi, Minami-ku, Hiroshima 734-8553, Japan; rfujimori@hiroshima-u.ac.jp (R.F.); tyutaka@hiroshima-u.ac.jp (Y.T.); kuwakuwaduck@hiroshima-u.ac.jp (N.K.); ue1320sc@hiroshima-u.ac.jp (S.U.); d250232@hiroshima-u.ac.jp (Y.H.); alphadentalplus@hiroshima-u.ac.jp (M.S.); tarimisa@hiroshima-u.ac.jp (M.T.); skono913@hiroshima-u.ac.jp (S.K.); iwatat@hiroshima-u.ac.jp (T.I.); mmiya@hiroshima-u.ac.jp (M.M.); mizuno@hiroshima-u.ac.jp (N.M.); 2 Department of Cardiovascular Medicine, Graduate School of Biomedical and Health Sciences, Hiroshima University, 1-2-3 Kasumi, Minami-ku, Hiroshima 734-8553, Japan; smiyauchi@hiroshima-u.ac.jp (S.M.); ynakano@xj8.so-net.ne.jp (Y.N.); 3 Division of Periodontology, Department of Regenerative Science in Conservative Dentistry, Kyushu Dental University, 2-6-1 Manazuru, Kokurakita-ku, Kitakyushu 803-8580, Japan; matsudas@hiroshima-u.ac.jp; 4 Center for Oral Clinical Examination, Hiroshima University, Hospital Hiroshima, 1-2-3 Kasumi, Minami-ku, Hiroshima 734-8553, Japan; tshintan@hiroshima-u.ac.jp (T.S.); mkajiya@hiroshima-u.ac.jp (M.K.)

**Keywords:** antithrombotic drugs, direct oral anticoagulants, periodontal disease, basic periodontal treatment, periodontal inflamed surface area, apixaban

## Abstract

**Background/Objectives**: The optimal management of periodontal treatment in patients receiving direct oral anticoagulants (DOACs) remains poorly established, and bleeding concerns may lead clinicians to delay or limit treatment. We evaluated the clinical efficacy and safety of nonsurgical periodontal treatment in patients receiving DOAC therapy. **Methods**: This retrospective study included 67 patients receiving continuous DOAC therapy (edoxaban, apixaban, dabigatran, or rivaroxaban) who underwent nonsurgical periodontal treatment without drug discontinuation. Pocket probing depth, bleeding on probing, the periodontal inflamed surface area (PISA), the periodontal epithelial surface area (PESA), and serum inflammatory cytokine levels were evaluated. A ligature-induced-periodontitis mouse model was also used to assess the effect of apixaban on alveolar bone loss. **Results**: Nonsurgical periodontal treatment significantly reduced the PISA (42.9%) and PESA (10.8%) in the overall DOAC cohort, with no postoperative bleeding complications. Significant reductions in periodontal parameters were seen in the edoxaban (PISA: 44.2%, PESA: 10.9% reduction), apixaban (PISA: 45.6%, PESA: 11.7% reduction), and dabigatran (PISA: 61.3%, PESA: 16.1% reduction) groups, with no significant changes in the rivaroxaban group. Baseline PISA was positively correlated with baseline serum levels of interleukin-4 (*r* = 0.33), interleukin-1 beta (*r* = 0.29), interleukin-6 (*r* = 0.25), and interleukin-10 (*r* = 0.30). In the mouse model, apixaban administration did not aggravate ligature-induced alveolar bone loss. **Conclusions**: Basic periodontal treatment appears to be safe and effective for patients prescribed DOACs and may reduce local periodontal inflammation, resulting in systemic inflammatory burden. These findings support proactive periodontal intervention in patients receiving DOAC therapy, regardless of the specific anticoagulant used.

## 1. Introduction

As populations age, the number of patients receiving anticoagulant therapy for various systemic conditions continues to increase. Direct oral anticoagulants (DOACs), including edoxaban, apixaban, dabigatran, and rivaroxaban, are widely prescribed to prevent thromboembolic events in patients with cardiovascular and thromboembolic diseases [1,2,3]. Additionally, periodontal disease is highly prevalent among older adults. Consequently, dentists are increasingly likely to encounter patients receiving anticoagulant therapy in daily clinical practice, and this has become an increasingly important clinical concern.

Current guidelines and previous studies have reported recommendations for managing invasive dental procedures, particularly tooth extraction, in patients receiving anticoagulant therapy [3,4,5,6]. In many cases, tooth extraction can be safely performed without discontinuation of DOACs when appropriate local hemostatic measures are applied. Therefore, unnecessary interruption of anticoagulant therapy is generally discouraged because of the potential associated risk of thromboembolic complications. However, despite the accumulated evidence regarding dental extraction, optimal management of periodontal treatment in patients receiving anticoagulant therapy remains insufficiently established [4,7]. Consequently, clinicians may adopt an overly conservative treatment approach in patients receiving anticoagulant therapy, such as limiting procedures to supragingival scaling or avoiding intensive subgingival instrumentation because of concern about intraoperative and postoperative bleeding complications. However, avoiding subgingival periodontal treatment for this reason may compromise infection and inflammation control, allowing periodontal inflammation to persist and potentially affect both oral and systemic health [8,9,10].

Following the establishment of tooth extraction guidelines in oral surgery, clear management standards are urgently needed for periodontal disease treatment as well. Unlike for tooth extraction, recommendations regarding periodontal treatment in patients receiving anticoagulant therapy remain limited. In periodontal treatment, removing biofilm from the tooth surface—a primary etiologic factor—is essential. However, some bleeding may occur during the procedure as instruments are used to access inflamed periodontal tissue [11,12,13]. There is a lack of consensus regarding the management of subgingival (intra-pocket) periodontal instrumentation, including scaling and root planing (SRP), in patients receiving anticoagulant therapy [4,7]. To our knowledge, no prior study has specifically combined the following three elements: direct oral anticoagulants (rather than vitamin K antagonists), nonsurgical periodontal treatment as the therapeutic intervention, and PISA/PESA as a quantitative measure of periodontal inflammatory burden; the present study was designed to address this gap. Although primary clinical studies directly examining periodontal treatment outcomes in anticoagulated patients remain scarce, a retrospective analysis of periodontal treatment performed under continued antithrombotic therapy and a recent prospective national survey addressing periprocedural DOAC management in periodontal surgery have reported that periodontal treatment-related bleeding can generally be controlled with local hemostatic measures [14,15]. Periodontitis is a chronic inflammatory disease associated with local tissue destruction and systemic inflammatory burden [10,16,17]. Persistent periodontal inflammation has been linked to diverse systemic conditions, including cardiovascular disease and diabetes mellitus, as well as adverse systemic outcomes [8,16,17]. Therefore, from both oral and systemic health perspectives, appropriate periodontal intervention in patients receiving anticoagulant therapy remains clinically important. This proactive clinical approach is well-justified by current multi-disciplinary guidelines for dental management in patients taking antithrombotic drugs. Accumulating clinical evidence demonstrates that unnecessary interruption or temporary discontinuation of DOACs poses a significantly higher risk of life-threatening thromboembolic events, such as ischemic stroke, compared to the minor risk of localized postoperative bleeding. As periodontal procedures, including SRP, allow for the immediate application of direct local hemostatic measures, providing these therapies without interrupting DOAC medication is considered both ethically and clinically rational.

Although clinical studies are essential for evaluating treatment safety and mucosal changes, they are inherently limited in their ability to elucidate the detailed tissue dynamics and potential radiographic or histological aggravation of bone structures under pharmacological influence. Therefore, it is rational to combine clinical observations with those inferred from standardized animal models. This will help determine whether factor Xa (FXa) inhibition by DOACs directly affects active, synchronized alveolar bone resorption under controlled inflammatory conditions.

Given these considerations, the primary aim of the present study was to retrospectively evaluate the clinical efficacy and safety of basic periodontal treatment in patients receiving continuous DOAC therapy in routine clinical practice, focusing on changes in conventional periodontal parameters, periodontal inflammatory surfaces, and bleeding-related complications. Furthermore, we aimed to translate and validate these findings biologically by investigating the specific effects of apixaban on alveolar bone loss using a ligature-induced periodontitis mouse model. We hypothesized that basic periodontal treatment would reduce periodontal inflammatory burden without increasing bleeding-related complications in patients receiving DOAC therapy and that apixaban would not exacerbate periodontal bone loss in the animal model. Overall, our findings suggest that basic periodontal treatment is safe and effective in patients receiving DOAC therapy, reduces periodontal inflammatory parameters, and may help reduce systemic inflammatory burden.

## 2. Materials and Methods

### 2.1. Clinical Research Participants

**Figure 1:**
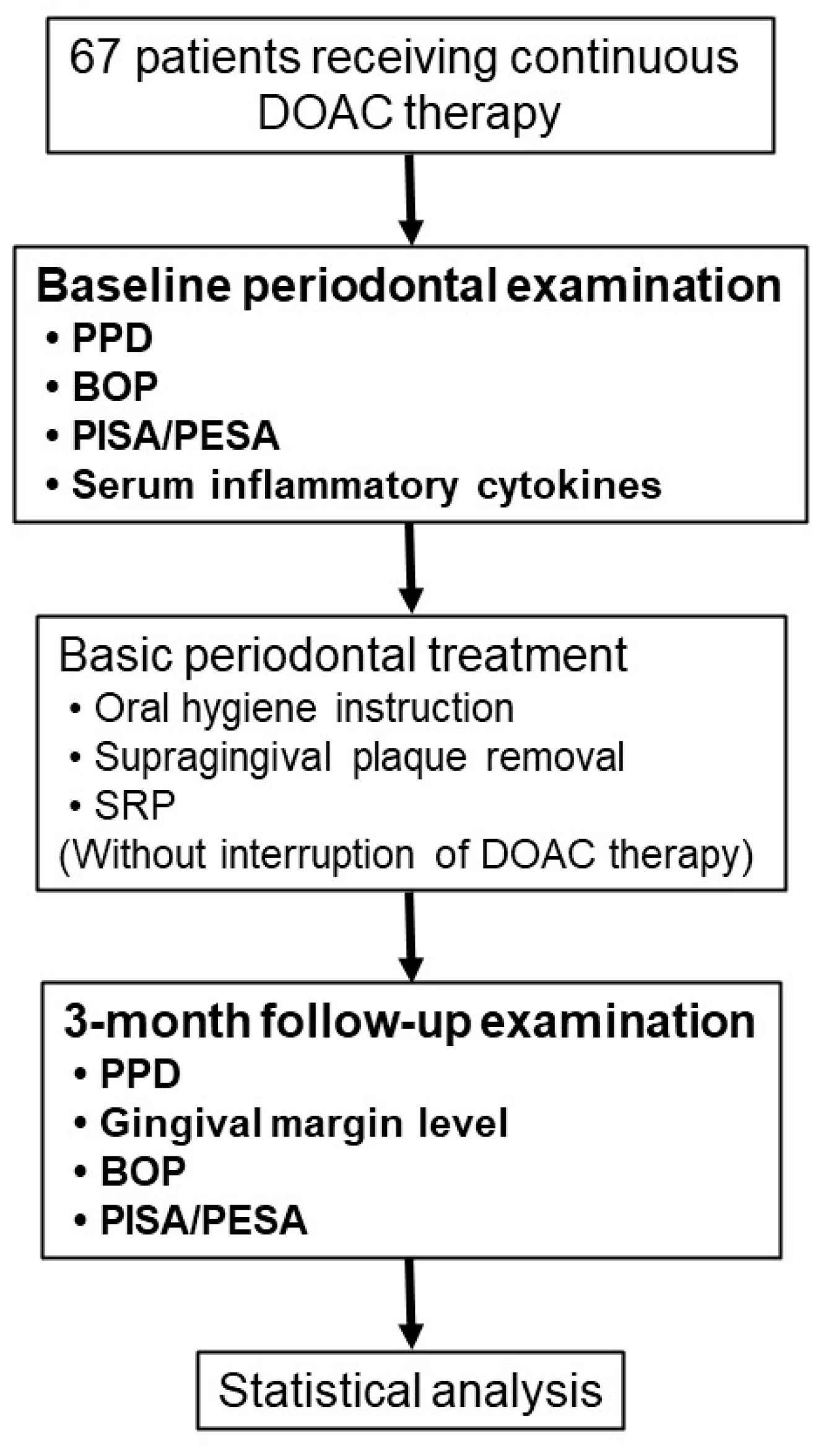
Schematic overview of the clinical study design. Clinical retrospective study workflow. A total of 67 patients receiving continuous direct oral anticoagulant (DOAC) therapy were evaluated. Clinical periodontal examinations—including pocket probing depth (PPD), bleeding on probing (BOP), Periodontal Inflamed Surface Area (PISA) and Periodontal Epithelial Surface Area (PESA)—and serum collection were performed at baseline. Patients received standard non-surgical basic periodontal treatment. Follow-up clinical re-evaluations and safety monitoring for bleeding-related complications were conducted 3 months post-treatment.

This retrospective study included 67 patients who were receiving continuous oral anticoagulant therapy and underwent periodontal treatment without interrupting their medication (Figure 1). Periodontal examinations were conducted before and after treatment, and the treatment protocol included supragingival plaque removal and SRP. Periodontal inflammatory status was assessed using the periodontal inflamed surface area (PISA) and periodontal epithelial surface area (PESA). Demographic and clinical data, including anticoagulant medication status, tobacco smoking history, alcohol consumption, and medical history, were extracted from medical records. Serum inflammatory cytokine levels were also assessed. Owing to the retrospective nature of the clinical portion of this study, a formal *a priori* sample size calculation was not performed, and all consecutive patients who met the selection criteria during the study period were included (n = 67). However, to confirm the statistical validity of our sample size, a post hoc power analysis was conducted for the primary outcome, which was the post-treatment reduction in periodontal inflamed surface area (ΔPISA). With a sample size of 67 patients and an alpha level of 0.05, the statistical power (1-beta) was calculated to be greater than 0.80, based on the observed effect size. This indicates that the sample size was sufficient to detect clinically meaningful changes in periodontal inflammatory parameters.

### 2.2. Periodontal Examination and Treatment

A single experienced dental clinician, blinded to the patients’ clinical information, performed comprehensive periodontal examinations using a periodontal probe (Hu-Friedy Mfg, Chicago, IL, USA). Pocket probing depth (PPD), gingival margin level, and bleeding on probing (BOP) were recorded at all periodontal sites. Periodontal inflammatory burden was calculated using PISA and PESA, as previously described [18]. After the baseline periodontal examination, all patients underwent basic periodontal treatment, which consisted of oral hygiene instruction, supragingival plaque removal, and SRP with ultrasonic scalers and Gracey curettes. Anticoagulant therapy was maintained throughout periodontal treatment. Patients were monitored for treatment-related complications, including prolonged postoperative bleeding, but no incidents were observed. Treatment effectiveness was evaluated through repeat periodontal examination, including PISA and PESA measurements, approximately 3 months after completion of periodontal treatment. To ensure safety and reporting objectivity, a “postoperative bleeding complication” was operationally defined as any episode of persistent, active bleeding from the treated periodontal sites that could not be controlled by standard initial compression (15 min) at the chairside, required advanced clinical interventions (e.g., the application of local hemostatic agents, cellulose sponges, or suturing) to achieve hemostasis, or involved secondary, spontaneous re-bleeding that occurred after the patient returned home and required emergency visits or re-evaluation. In this cohort, hemostasis was achieved in all cases through local compression. Suturing was prepared as an additional measure to address refractory bleeding, rather than being a procedure routinely required for SRP (scaling and root planing) itself. This stepwise approach to local hemostasis is consistent with previously reported findings regarding periodontal treatment for patients undergoing antithrombotic therapy [14,19]. Patients were monitored at specific time points: immediately after the completion of subgingival instrumentation (chairside for 15 min), before leaving the clinic, and via clinical evaluation at all subsequent appointment intervals. No such treatment-related prolonged or secondary bleeding complications were observed during the monitoring periods across the entire 67-patient cohort.

### 2.3. Measurement of Inflammatory Cytokines

With each patient’s consent, residual serum samples obtained during routine blood testing while patients were receiving anticoagulant therapy were used for these analyses. Approval for serum use was obtained from the Hiroshima University Clinical Research Ethics Committee (approval number E2023-0032). Serum samples obtained at the baseline periodontal examination were used to measure inflammatory cytokines and chemokines. Cytokine levels were quantified using LEGENDplex (LEGENDplex™ HU Essential Immune Response Panel (13-plex) #740930; BioLegend, San Diego, CA, USA) according to the manufacturer’s instructions and analyzed using a FACSVerse flow cytometer (BD Biosciences; Franklin Lakes, NJ, USA). The target analytes and their minimum detectable concentrations were: interleukin (IL)-4, 0.86 pg/mL; IL-2, 1.53 pg/mL; CXCL10 (IP-10), 1.32 pg/mL; IL-1β, 0.65 pg/mL; tumor necrosis factor α, 0.97 pg/mL; C-C motif chemokine ligand 2 (MCP-1), 1.45 pg/mL; IL-17A, 1.41 pg/mL; IL-6, 0.86 pg/mL; IL-10, 1.02 pg/mL; IFN-γ, 0.79 pg/mL; IL-12p70, 0.88 pg/mL; CXCL8 (IL-8), 0.90 pg/mL; and transforming growth factor β1, 3.06 pg/mL.

### 2.4. Experimental Periodontitis Mouse Model

All animal procedures were approved by the Ethics Committee of Hiroshima University (approval No. A25-110). A total of 24 male C57BL/6J Jcl mice (8 weeks old; CLEA Japan, Inc., Tokyo, Japan) were housed under specific pathogen-free conditions in a controlled environment (temperature 22–24 °C, relative humidity 40–60%) under a 12 h light/dark cycle. The mice were fed gamma-irradiated regular chow (Type MF; Oriental Yeast Co., Ltd., Tokyo, Japan), and all animal experiments were conducted in accordance with the ARRIVE guidelines (https://arriveguidelines.org/arrive-guidelines; accessed on 12 December 2021). Experimental periodontitis was induced using a ligature model. Mice were randomly assigned to four groups: control, apixaban-treated, ligature, and ligature with apixaban treatment (n = 6 per group). The sample size was determined using an *a priori* power analysis based on previous data [20]. With an alpha level of 0.05 and a power of 0.80, a sample size of six mice per group was determined to be sufficient to detect significant differences in alveolar bone loss.

Apixaban (50 μg) was administered orally, twice daily, for 2 weeks prior to ligature placement and administration was continued throughout the duration of the experiment. FXa inhibition was determined by using SensoLyte Factor Xa Assay Kit (AnaSpec, Inc., Fremont, CA, USA) before the experiments. Ligatures were placed around the maxillary second molars to induce experimental periodontitis and were maintained for 5 days while apixaban administration continued. A 5-day ligature period was selected because it reflects a widely used protocol for capturing the acute, synchronized phase of inflammatory alveolar bone resorption in mice, allowing bone loss to be reliably detected while minimizing ligature loss or spontaneous displacement over longer periods. Apixaban was co-administered throughout this period to test the hypothesis that FXa inhibition, through its potential effects on protease-activated receptor signaling and local inflammatory pathways, might modulate the acute inflammatory bone resorptive response to periodontal pathogenic challenge; this rationale and its limitations for capturing chronic bone remodeling are discussed further below. Following the experimental period, the mice were euthanized by carbon dioxide inhalation followed by cervical dislocation [20]. No animals or data points were excluded from the analyses, as no complications or premature deaths occurred during the experimental period. Alveolar bone loss was then assessed using micro-computed tomography (micro-CT). Detailed image acquisition and analysis procedures are described below.

### 2.5. Micro-CT Analysis

Maxillary specimens were scanned using a micro-CT system (Skyscan 1076; Bruker, Kontich, Belgium). Micro-CT imaging and subsequent bone loss quantification were performed by a single investigator who was blinded to the experimental group assignments to prevent bias. The acquired images were reconstructed and exported in bitmap format. Reconstructed images were oriented along standardized horizontal and vertical planes using DataViewer software (version 1.4.4, 64-bit; Bruker, Kontich, Belgium). Alveolar bone loss was quantified using CTAn software (version 1.12.0.0+; Bruker, Kontich, Belgium). For the sagittal sections of the maxillary second molars, slices showing identifiable root apices of the mesiobuccal, distobuccal, and palatal roots were selected for analysis. The distances from the cementoenamel junction to the alveolar bone crest were measured at each root site, and these values were summed for each specimen. Group means were then compared among the experimental groups. For three-dimensional reconstruction, regions of interest were defined in CTAn, and bone tissue was segmented and reconstructed using CTVol software (version 2.2.1.0, 64-bit; Bruker, Kontich, Belgium). Representative three-dimensional images were generated for the left maxillary second-molar region.

### 2.6. Statistical Analysis

Continuous variables were summarized as mean ± standard deviation or median (interquartile range), as appropriate. Data normality was assessed using the Shapiro–Wilk test. Paired comparisons were performed using the Wilcoxon signed-rank test. Comparisons between two groups were conducted using the Mann–Whitney U test, whereas comparisons among multiple groups were performed using the Kruskal–Wallis test. Correlations between continuous variables were evaluated using Spearman’s rank correlation coefficient. Multivariable linear regression analysis was performed to identify factors associated with changes in periodontal inflamed surface area (ΔPISA). In the experimental periodontitis mouse model, alveolar bone loss was compared among the four experimental groups using one-way analysis of variance, followed by Tukey’s multiple comparison test. Effect sizes for clinical pre- and post-treatment comparisons were evaluated using Cohen’s d. A *p*-value < 0.05 was considered to indicate statistical significance. All statistical analyses were performed using EZR software (version 4.5.2; Saitama Medical Center, Jichi Medical University, Saitama, Japan).

## 3. Results

### 3.1. Patient Characteristics

Table 1 summarizes the characteristics of the 67 patients who received continuous oral anticoagulant therapy. The mean age was 67.3 ± 10.3 years, and 64.2% of patients were male. Edoxaban was the most frequently prescribed DOAC (34.3%), followed by apixaban (28.4%), dabigatran (19.4%), and rivaroxaban (17.9%).

### 3.2. Periodontal Treatment Outcomes

**Figure 2:**
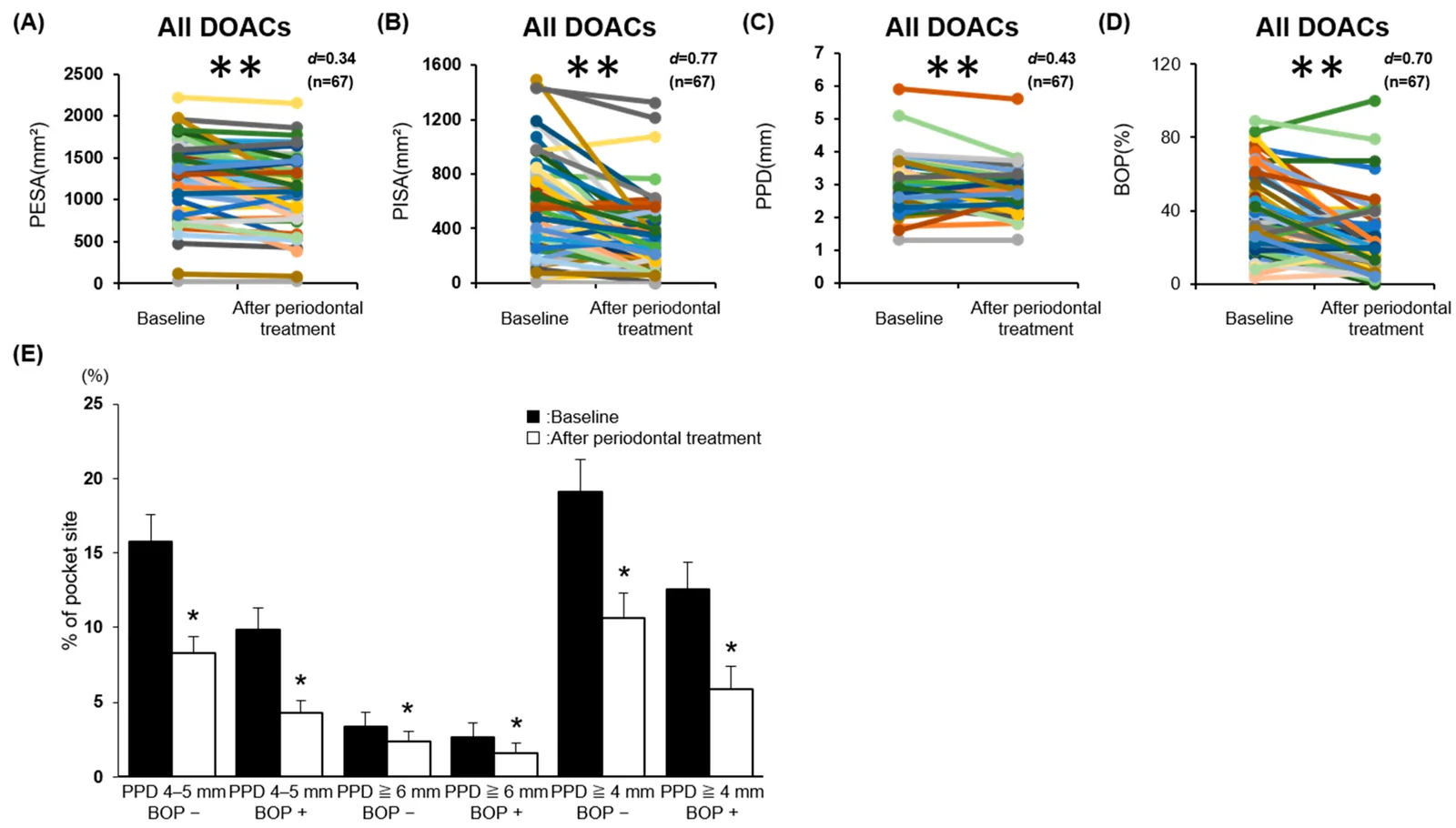
Changes in periodontal inflammatory parameters after basic periodontal treatment in patients receiving direct oral anticoagulant (DOAC) therapy. (**A**–**D**) Overall DOAC cohort. Periodontal epithelial surface area (PESA, (**A**)), periodontal inflamed surface area (PISA, (**B**)), pocket probing depth (PPD, (**C**)), bleeding on probing (BOP, (**D**)), and change in the proportion of pockets with a depth of 4 mm or more (**E**) values before and after periodontal treatment were compared using the Wilcoxon signed-rank test. Each colored line connects the values before and after periodontal treatment for an individual patient. * *p* < 0.05, ** *p* < 0.01.

**Figure 3:**
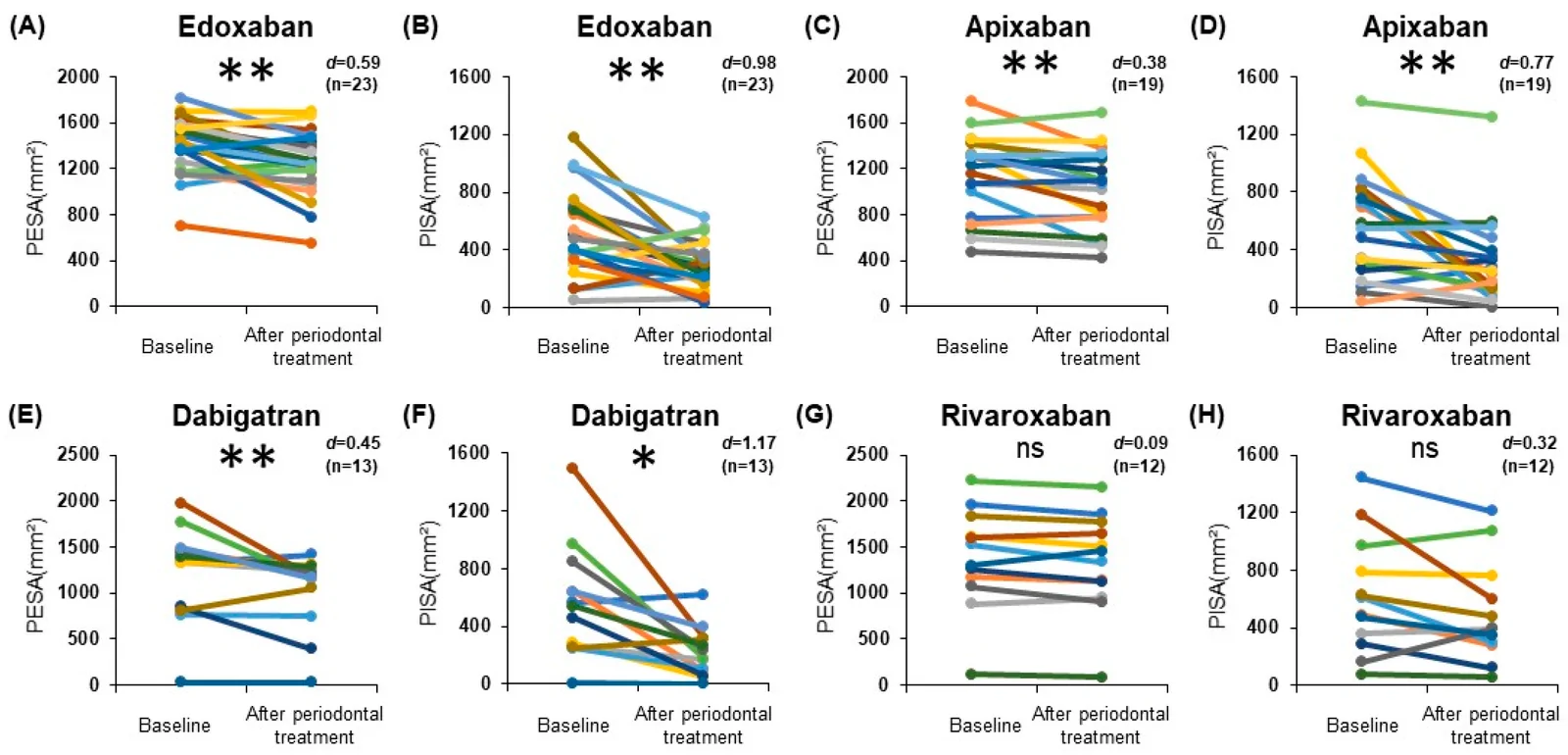
Changes in periodontal inflammatory parameters after basic periodontal treatment in patients receiving direct oral anticoagulant (DOAC) therapy. (**A**,**B**) Edoxaban group. (**C**,**D**) Apixaban group. (**E**,**F**) Dabigatran group. (**G**,**H**) Rivaroxaban group. Periodontal epithelial surface area (PESA, (**A**,**C**,**E**,**G**)) and periodontal inflamed surface area (PISA, (**B**,**D**,**F**,**H**)) values before and after periodontal treatment were compared using the Wilcoxon signed-rank test. Each colored line connects the values before and after periodontal treatment for an individual patient. ns, not significant; * *p* < 0.05, ** *p* < 0.01.

Basic periodontal treatment significantly reduced PISA and PESA in the overall DOAC cohort (mean reduction rates: 42.9% for PISA and 10.8% for PESA; Figure 2A and B), showing clinically meaningful effect sizes (PISA: Cohen’s *d* = 0.77; PESA: Cohen’s *d* = 0.35). In addition, significant improvements were observed in conventional periodontal parameters, with the mean PPD reduced by 9.6% and BOP reduced by 37.7% (Figure 2C and D), corresponding to moderate and moderate-to-large effect sizes, respectively (PPD: Cohen’s *d* = 0.43; BOP: Cohen’s *d* = 0.71). We also focused on periodontal pockets of depth 4 mm or greater and compared their reduction rates. Regardless of BOP status, a decrease in prevalence was observed following basic periodontal therapy across all categories: 4–5 mm, 6 mm or greater, and 4 mm or greater (Figure 2E). When PISA and PESA reductions were analyzed by DOAC type, similar decreases were observed in the edoxaban (PISA: 44.2% reduction; PESA: 10.9% reduction), apixaban (PISA: 45.6% reduction; PESA: 11.7% reduction), and dabigatran (PISA: 61.3% reduction; PESA: 16.1% reduction) groups (Figure 3A–F). The rivaroxaban group also showed a non-significant decrease in periodontal inflammatory parameters (Figure 3G, H).

### 3.3. Association Between Periodontal Inflammation and Systemic Cytokines

**Figure 4:**
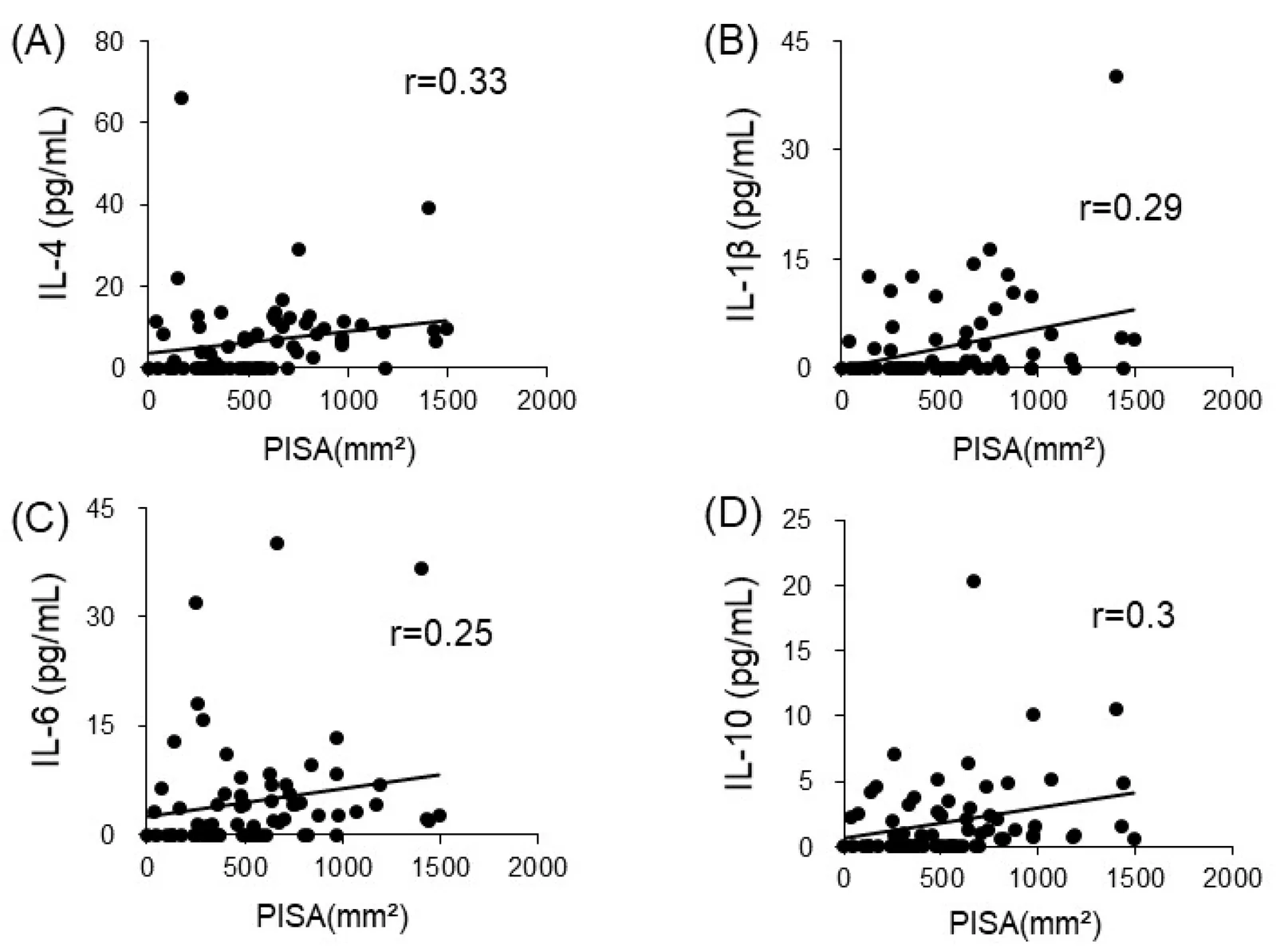
Correlations between baseline periodontal inflamed surface area (PISA) and systemic inflammatory cytokine levels. Correlations between baseline PISA and serum inflammatory cytokine levels were assessed using Spearman’s rank correlation coefficient. Significant positive correlations were observed between baseline PISA and serum levels of interleukin (IL)-4 (**A**), IL-1β (**B**), IL-6 (**C**), and IL-10 (**D**).

We evaluated correlations between the baseline periodontal inflammatory burden and systemic inflammatory cytokine levels before periodontal treatment (Figure 4). Significant positive correlations were observed between baseline PISA and IL-4 (Figure 4A, *r* = 0.33, *p* = 0.007), IL-1β (Figure 4B, *r* = 0.29, *p* = 0.017), IL-6 (Figure 4C, *r* = 0.25, *p* = 0.038), and IL-10 (Figure 4D, *r* = 0.30, *p* = 0.013). As serum cytokine levels were measured only at baseline in this retrospective cohort, no post-treatment cytokine data were available; therefore, changes in cytokine levels after periodontal treatment could not be evaluated in the present study.

### 3.4. Factors Associated with Periodontal Treatment Response

To identify factors associated with periodontal treatment response, post-treatment changes in PISA (ΔPISA) were analyzed. Associations between patient characteristics and periodontal treatment response were evaluated using ΔPISA values. No significant differences in ΔPISA were observed according to sex, diabetes mellitus, hypertension, tobacco smoking history, or alcohol consumption. Similarly, periodontal treatment responses did not differ significantly among the DOAC groups. Multivariable linear regression analysis showed that baseline PISA was independently associated with ΔPISA, whereas age, sex, diabetes mellitus, and smoking history were not significantly associated with periodontal treatment responses.

### 3.5. Effect of Apixaban Administration on Alveolar Bone Loss in an Experimental Periodontitis Mouse Model

**Figure 5:**
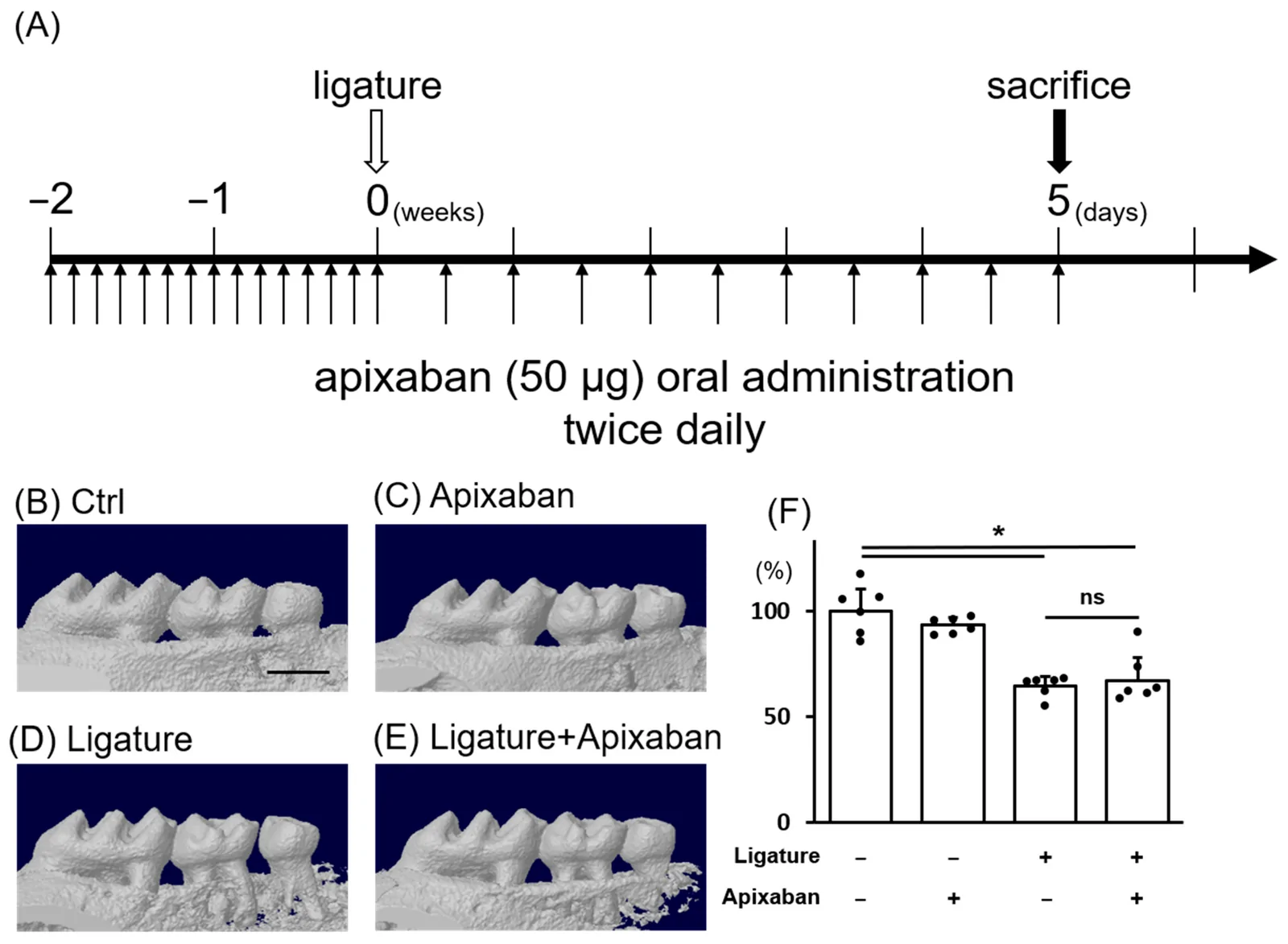
Effect of apixaban administration on alveolar bone loss in the ligature-induced periodontitis model. In (**A**), the open and filled arrows indicate ligature placement and sacrifice, respectively. Experimental schedule for the generation of a mouse periodontitis model (**A**). Representative micro-computed tomography (micro-CT) images showing alveolar bone morphology in the control (**B**), apixaban-treated (**C**), ligature (**D**), and ligature-with-apixaban-treatment (**E**) groups (n = 6 mice per group). Apixaban (50 μg) was administered orally twice daily, starting 2 weeks before ligature placement, and continued throughout the experimental period. The bar in the lower right indicates 500 μm (**B**). Alveolar bone loss was quantified using reconstructed micro-CT images (**F**). Data are presented as mean ± standard deviation. * *p* < 0.05; ns, not significant.

To investigate the possible effects of anticoagulant therapy on periodontal tissue destruction, alveolar bone loss was assessed in a ligature-induced periodontitis mouse model (Figure 5). Representative micro-CT images showed greater alveolar bone loss in the ligature-induced periodontitis groups (ligature group) than in the control groups (Figure 5B–E). Quantitative analysis showed significant differences in alveolar bone loss among the four experimental groups (one-way analysis of variance, *p* < 0.001). Post hoc Tukey analysis showed significantly greater alveolar bone loss in both the ligature and ligature with apixaban groups than in the control and apixaban-only groups. However, alveolar bone loss did not differ significantly between the ligature and ligature with apixaban groups. Apixaban administration alone also did not significantly affect alveolar bone loss compared with the control group (Figure 5F).

## 4. Discussion

In the present study, we evaluated the clinical efficacy and safety of basic periodontal treatment in patients receiving DOAC therapy. Our clinical findings showed that basic periodontal treatment significantly reduced both PISA and PESA across the overall DOAC cohort without postoperative hemorrhagic complications or systemic adverse events. These findings suggest that standard basic periodontal treatment may be performed safely and effectively in patients receiving long-term DOAC therapy when appropriate local hemostatic measures are used. The justification for continuing DOAC therapy during non-surgical periodontal intervention is rooted in balancing the systemic thromboembolic risk against local hemorrhagic risk. Although explicit, universal consensus guidelines dedicated specifically to subgingival periodontal instrumentation remain limited compared to oral surgery guidelines, available evidence specifically addressing subgingival periodontal instrumentation, rather than tooth extraction or oral surgery, in anticoagulated patients is comparatively limited to a small number of retrospective series and surveys, several of which predate the widespread clinical use of DOACs [14,15,19]. Our retrospective design reflects a real-world clinical strategy that prioritizes the prevention of thromboembolism. By demonstrating a complete absence of prolonged or secondary bleeding complications in our 67-patient cohort, we provide strong empirical justification for the safety of performing comprehensive subgingival debridement without drug discontinuation.

Furthermore, correlation analysis showed that baseline PISA was positively correlated with serum levels of IL-4, IL-1β, IL-6, and IL-10. These results suggest that the baseline severity of local periodontal inflammation may reflect systemic inflammatory status in patients taking DOACs [9,21,22]. However, because serum cytokine levels were assessed only at baseline and not after treatment, this study cannot directly demonstrate a treatment-related reduction in systemic inflammatory burden. Based on this baseline association alone, we hypothesize, rather than conclude, that the post-treatment decrease in PISA and PESA may parallel a reduction in systemic inflammatory burden; this hypothesis will require confirmation through longitudinal cytokine measurement in future prospective studies, which may be relevant to cardiovascular and other systemic risks associated with chronic inflammation [23,24].

It should also be noted that, although statistically significant, the correlations between baseline PISA and serum IL-4, IL-1β, IL-6, and IL-10 were weak in magnitude (r = 0.25–0.33), so baseline PISA accounted for only a small proportion of the variance in cytokine levels. Such weak correlations are not unexpected given that circulating cytokine concentrations are influenced by multiple systemic and lifestyle factors beyond the local periodontal inflammatory burden, and that a single periodontal index cannot be expected to fully capture systemic immune status. We therefore interpret these associations as indicating a modest but biologically plausible link between local periodontal inflammation and systemic cytokine levels, rather than as evidence of a strong or clinically predictive relationship.

When outcomes were analyzed by DOAC type, significant reductions in PISA and PESA were observed in the edoxaban, apixaban, and dabigatran groups. In contrast, the rivaroxaban group did not show significant pre- to post-treatment differences. This discrepancy may be attributed to the relatively small sample size (n = 12) of the rivaroxaban group and individual variations among patients. Additionally, the pharmacological characteristics of rivaroxaban, including its once-daily dosing regimen, may have contributed to greater clinical variability in adherence or trough levels compared with the twice-daily regimens of apixaban and dabigatran [25]. Because edoxaban, apixaban, and dabigatran achieve anticoagulation through closely related mechanisms (direct factor Xa inhibition for edoxaban and apixaban, and direct thrombin inhibition for dabigatran, both converging on the terminal steps of the coagulation cascade), we do not consider it biologically plausible that the drug itself directly caused differential periodontal treatment responses among these three agents. Rather, we interpret the between-group differences in this study as most likely reflecting differences in sample size, dosing frequency, and pharmacokinetic variability rather than a true anticoagulant-specific effect on periodontal healing. Alternatively, this lack of significance may reflect limited statistical power because of the small sample size. This represents a limitation of the present study, and further validation in larger multicenter collaborative studies is needed.

To identify factors independently associated with periodontal treatment response, we performed a multivariable linear regression analysis. Baseline PISA was the only factor independently associated with ΔPISA, whereas age, sex, diabetes mellitus, and smoking history were not significantly associated with this outcome. This finding has important clinical implications as it suggests that, even in patients receiving anticoagulant therapy, periodontal improvement is influenced primarily by the initial severity of local inflammation, as reflected by baseline PISA, rather than by systemic factors, such as diabetes mellitus. Therefore, standard basic periodontal treatment should be considered for patients receiving DOAC therapy when their systemic conditions are adequately controlled, particularly because patients with more severe baseline inflammation may benefit the most [9]. The mean baseline PPD in this study was 2.9 mm, which falls within a clinically acceptable low-risk range. However, it included localized areas with severe destruction, where the maximum PPD reached 12 mm. Relying solely on conventional site-specific mean values often dilutes the clinical significance of these localized severe lesions, masking true periodontal inflammation. A detailed analysis of deep pockets revealed significant improvements following basic periodontal therapy across the categories of 4–5 mm, ≥6 mm, and ≥4 mm depth—regardless of BOP status—indicating that the periodontal treatment is progressing effectively (Figure 2E). To overcome these limitations of conventional PPD mean values and accurately quantify the total inflammatory surface area of active periodontitis, we used PISA. PISA integrates both pocket depth and circumferential extent; therefore, it allows for a more comprehensive and realistic assessment of systemic inflammation risk originating from oral tissues, even when the mean PPD value appears unremarkable.

Furthermore, animal experiments using a ligature-induced periodontitis model showed that oral administration of apixaban did not aggravate alveolar bone loss under non-ligated control or periodontitis conditions. This suggests that apixaban-mediated FXa inhibition does not appear to promote periodontal tissue destruction. Beyond this safety finding, emerging evidence suggests that FXa inhibition may modulate inflammation and bone metabolism through protease-activated receptor pathways [26,27]. Although apixaban did not significantly reduce bone loss in comparison to the periodontitis model in this study, the absence of exacerbated bone loss supports the biological safety of FXa inhibition in inflammatory periodontal bone destruction and strengthens the translational relevance of the clinical findings.

A few limitations of this study should be acknowledged. First, this was a retrospective study conducted at a single center, and it lacked a control group of patients with periodontitis not taking anticoagulants under the same treatment protocol. Therefore, we cannot directly compare the magnitude of the 42.9% PISA reduction with outcomes in the standard population. Our primary comparison was therefore within-subject (pre- versus post-treatment), which controls for each patient acting as their own baseline but cannot fully disentangle the effect of periodontal treatment itself from that of continued anticoagulant use. Because BOP and PPD are generally expected to improve after nonsurgical periodontal treatment regardless of anticoagulant status, a prospective study incorporating a non-anticoagulated comparator group, ideally matched for periodontal disease severity, would be needed to determine whether the magnitude of improvement observed here differs from that expected in the general periodontitis population. Second, in the experimental periodontitis mouse model, the 5-day duration of ligature placement under apixaban administration might be too short to fully evaluate long-term bone remodeling or chronic bone loss aggravation. Future prospective, multi-center studies with larger sample sizes and extended timelines for animal experiments are warranted to validate these findings. Furthermore, a minor limitation regarding the translational component involves the experimental periodontitis mouse model. In this study, the ligature was maintained for 5 days, which is a widely accepted standard protocol for capturing the acute, synchronized phase of inflammatory alveolar bone resorption. However, this short duration may not be sufficient to evaluate the long-term, chronic effects of continuous FXa inhibition on broader bone remodeling and turnover. Although our micro-CT data confirmed that apixaban did not exacerbate initial bone destruction, the lack of an extended observational period limits our understanding of chronic bone metabolism under DOAC therapy, which should be addressed in future long-term animal studies. A more extended protocol, such as maintaining the ligature for at least 14 days followed by ligature removal and observation of continued resorption or healing, would provide a more rigorous assessment of chronic, rather than purely acute, bone loss dynamics under continuous FXa inhibition [28]. A related limitation is that our ligature model did not incorporate deliberate inoculation with human periodontal pathogens; ligature-alone models can produce alveolar bone defects that are less stable than those induced by ligature combined with a defined periodontal pathogen challenge, and future studies should consider a humanized, pathogen-supplemented ligature model to more closely approximate the microbial etiology of human periodontitis and to establish a more stable and clinically translatable periodontal defect [28]. Additionally, another notable limitation is the lack of longitudinal serum cytokine data. As the primary outcome of this retrospective study was the clinical and surface area reduction in local periodontal inflammation (PISA), blood sampling was performed only at baseline to evaluate the initial systemic inflammatory burden associated with periodontitis. Consequently, the effect of non-surgical periodontal treatment on the direct reduction in systemic inflammatory markers could not be longitudinally tracked in this cohort, which should be investigated in future prospective studies. The 13-plex cytokine panel used in this study was selected primarily because it was the pre-validated, commercially available multiplex assay established in our laboratory for characterizing the general pro-inflammatory, anti-inflammatory, and Th-subset cytokine/chemokine profile associated with periodontal inflammation, based on our prior work in periodontitis-related systemic inflammation. We acknowledge that, given the specific focus of this study on bleeding risk and local wound healing under anticoagulant therapy, components of the fibrinolytic and coagulation systems (e.g., plasmin/plasminogen-related mediators), which are directly linked to clot stability, extracellular matrix remodeling, and neutrophil activation in the periodontal microenvironment, would have been a more mechanistically targeted choice and were not included in the present panel [29]. Serum, rather than saliva or gingival crevicular fluid (GCF), was used because residual samples from routine clinical blood testing performed while patients were receiving anticoagulant therapy were readily available for this retrospective analysis and reflect systemic inflammatory status; we recognize that saliva or GCF may better capture the local periodontal inflammatory microenvironment, and prospective collection of these fluids is planned for future work.

## 5. Conclusions

Our results suggest that basic periodontal treatment is safe and effective for patients prescribed DOACs, and it leads to significant reductions in periodontal clinical parameters. Moreover, our findings suggest that local periodontal control may help reduce systemic inflammatory markers in this population. Although further large-scale multicenter studies are needed to confirm these findings and clarify differences in outcomes among individual DOACs, our results provide clinically relevant evidence supporting proactive periodontal disease management in patients receiving DOAC therapy.

## Figures and Tables

**Table 1:** Patient characteristics.

Variable	Total (n = 67)	
Age, years	67.3 ± 10.3	
Male, n (%)	43 (64.2)	
Hypertension, n (%)	46 (68.7)	
Diabetes mellitus, n (%)	8 (11.9)	
Dyslipidemia, n (%)	35 (52.2)	
Ever smoker, n (%)	37 (55.2)	
Current alcohol consumption, n (%)	38 (56.7)	
DOAC type, n (%)	Edoxaban	23 (34.3)
	Apixaban	19 (28.4)
	Dabigatran	13 (19.4)
	Rivaroxaban	12 (17.9)
Baseline PISA, mm^2^	501.5 (296.9–736.2)	
Baseline PESA, mm^2^	1339.3 (1110.9–1537.3)	
After periodontal treatment PISA, mm^2^	266.7 (134.3–393.6)	
After periodontal treatment PESA, mm^2^	1216.2 (972.6–1370.1)	
Baseline PPD, mm	2.9 (2.5–3.3) (single PPD, max: 12 mm, min: 1 mm)	
After periodontal treatment PPD, mm	2.6 (2.3–2.9) (single PPD, max: 10 mm, min: 1 mm)	
Baseline BOP, %	32 (22–43.5)	
After periodontal treatment BOP, %	18 (12–29.2)	
ΔPISA, mm^2^ (Post-treatment reduction)	195 (12.4–396.8)	

Data are presented as mean ± SD, median (IQR), or n (%), as appropriate. Abbreviations: DOAC, direct oral anticoagulant; PISA, periodontal inflamed surface area; PESA, periodontal epithelial surface area; SD, standard deviation; IQR, interquartile range; PPD, probing pocket depth; BOP, bleeding on probing.

## Data Availability

All data generated or analyzed during this study are included in this published article.

## Abbreviations

The following abbreviations are used in this manuscript:

ARRIVE	Animal Research: Reporting of In Vivo Experiments
BOP	Bleeding on probing
DOACs	Direct oral anticoagulants
FXa	Factor Xa
IL	Interleukin
micro-CT	Micro-computed tomography
PPD	Pocket probing depth
PESA	Periodontal epithelial surface area
PISA	Periodontal inflamed surface area
SRP	Scaling and root planing
